# Integration Host Factor (IHF) binds to the promoter region of the *phtD *operon involved in phaseolotoxin synthesis in *P. syringae *pv. phaseolicola NPS3121

**DOI:** 10.1186/1471-2180-11-90

**Published:** 2011-05-04

**Authors:** Jackeline Lizzeta Arvizu-Gómez, Alejandro Hernández-Morales, Guillermo Pastor-Palacios, Luis G Brieba, Ariel Álvarez-Morales

**Affiliations:** 1 Departamento de Ingeniería Genética, Centro de Investigación y de Estudios Avanzados del Instituto Politécnico Nacional Unidad Irapuato, Apdo Postal 629, CP 36821, Irapuato, Gto, México; 2 Laboratorio Nacional de Genómica para la Biodiversidad, Centro de Investigación y de Estudios Avanzados del Instituto Politécnico Nacional, Apdo Postal 629, CP 36821, Irapuato, Gto, México

## Abstract

**Background:**

*Pseudomonas syringae *pv. phaseolicola, the causal agent of halo blight disease in beans, produces a toxin known as phaseolotoxin, in whose synthesis participate a group of genes organized within the genome in a region known as the "Pht cluster". This region, which is thought to have been acquired by horizontal gene transfer, includes 5 transcriptional units, two monocistronic (*argK, phtL*) and three polycistronic (*phtA, phtD, phtM*), whose expression is temperature dependent. So far, the regulatory mechanisms involved in phaseolotoxin synthesis have not been elucidated and the only well-established fact is the requirement of low temperatures for its synthesis. In this work, we searched for regulatory proteins that could be involved in phaseolotoxin synthesis, focusing on the regulation of the *phtD *operon.

**Results:**

In this study we identified the global regulator IHF (Integration Host Factor), which binds to the promoter region of the *phtD *operon, exerting a negative effect on the expression of this operon. This is the first regulatory protein identified as part of the phaseolotoxin synthesis system. Our findings suggest that the Pht cluster was similarly regulated in the ancestral cluster by IHF or similar protein, and integrated into the global regulatory mechanism of *P. syringae *pv. phaseolicola, after the horizontal gene transfer event by using the host IHF protein.

**Conclusion:**

This study identifies the IHF protein as one element involved in the regulation of phaseolotoxin synthesis in *P. syringae *pv. phaseolicola NPS3121 and provides new insights into the regulatory mechanisms involved in phaseolotoxin production.

## Background

*Pseudomonas syringae *pv. phaseolicola is a pathogenic bacterium, that produces a disease in beans (*Phaseolus vulgaris *L.) known as "Halo Blight". This disease affects both leaves and pods, and is responsible for major field crop losses in temperate areas. Disease symptoms are typically water-soaked lesions surrounded by a chlorotic zone or halo. This halo is due to the action of a non-host specific toxin known as phaseolotoxin [N^δ^(N'-sulfodiaminophosphinyl)-ornithyl-alanyl-homoarginine], which is the major virulence factor of the pathogen and a key component in the development of the disease [1-3]. Phaseolotoxin acts as a reversible inhibitor of the enzyme ornithine carbamoyltransferase (OCTase; EC2.1.3.3) that catalyzes the conversion of ornithine to citruline in the arginine biosynthesis pathway [4,5]. The consequence of OCTase inhibition is blockage of arginine biosynthesis resulting in death of host cells. The production of phaseolotoxin by *P. syringae *pv. phaseolicola is regulated by temperature, being optimally produced at 18°C-20°C, while at 28°C (the optimal growth temperature for this bacterium) the toxin is not detected [6,7]. Nevertheless, other factors such as plant signals and carbon sources have also been suggested as inducers of phaseolotoxin synthesis [8,9].

Our group reported the sequence of a chromosomal region of *P. syringae *pv. phaseolicola NPS3121, which contains genes involved in phaseolotoxin synthesis. This region, known as the "Pht cluster", includes 23 genes organized in five transcriptional units: two monocistronic, *argK *and *phtL*, and three polycistronic, a large operon from *phtA *to *phtK*, with an internal promoter capable of driving expression of *phtD *to *phtK *and a third operon that includes genes from *phtM *to *phtV *[10]. The function of *argK, desI, amtA *and *phtU *is known, while the function of the remaining genes remains uncertain [11-15]. The Pht cluster is also present in other phaseolotoxin-producing pathovars, including *P. syringae *pv. actinidiae (a kiwi pathogen) and in a single strain of *P. syringae *pv. syringae CFBP3388, although in the latter the cluster organization is poorly conserved [16,17]. Different evidence has suggested that the Pht cluster was acquired in these pathovars by horizontal gene transfer, most likely from a Gram positive bacterium [18-20]. However, whether this cluster contains all the elements necessary for phaseolotoxin production is still unknown.

Analysis of gene expression within the Pht cluster showed that most of the genes are transcribed at high levels at 18°C with a basal level of expression at 28°C, which agrees with the observed temperature-dependent pattern of phaseolotoxin synthesis, with the exception of *phtL*, which was expressed at both temperatures [10]. The mechanism by which *P. syringae *pv. phaseolicola regulates the expression of these genes in relation to temperature is poorly understood. Analysis of the promoter regions identified in the Pht cluster showed that the divergent promoters for *argK *and *phtA *contain canonic sequences of σ^70^-type promoters, while the promoter regions for *phtD, phtL *and *phtM *did not show similarity to consensus sequences for bacterial sigma factors. However, a common mechanism of transcriptional regulation for *phtD *and *phtM *has been suggested due to the presence of conserved regions in the promoters of these operons. Furthermore, analysis of transcriptional fusions of the Pht cluster promoter regions suggest that temperature regulation occurs at the transcriptional level since maximal transcriptional activity occurs at 18°C and is significantly lower at 28°C [10].

In bacteria, transcriptional regulation is commonly mediated by regulatory proteins that control gene expression in response to internal metabolic changes or external signals such as temperature, pH, and carbon source [21,22]. Previous reports proposed that *argK *regulation is under negative control mediated by a repressor protein present at 28°C, although the identity of this regulatory protein has not been elucidated [23]. Similarly, a regulatory function for the PhtL protein has been suggested based on the lack of *phtM *operon expression in a *phtL^- ^*background, although this still requires experimental confirmation [10]. Despite our knowledge of the effect of low temperature on phaseolotoxin synthesis, the regulatory mechanisms that control toxin production remain poorly understood. So far it is not known whether all the genes involved in the regulation of phaseolotoxin synthesis are located within the Pht cluster, or whether there are any other genes outside the Pht cluster involved in this process. In the latter case, it would be interesting to know whether any regulatory gene found outside the Pht cluster is specifically required for phaseolotoxin synthesis, or whether the synthesis of the toxin has adapted its expression to the regulatory mechanisms of the bacteria during horizontal gene transfer. For these reasons, this study was undertaken with the objective of identifying regulatory proteins that could participate in the regulation of genes for phaseolotoxin synthesis, with a focus on the regulation of the *phtD *operon.

## Results

### The promoter region of the *phtD *operon contains a binding site for a putative regulatory protein

**Figure 1:**
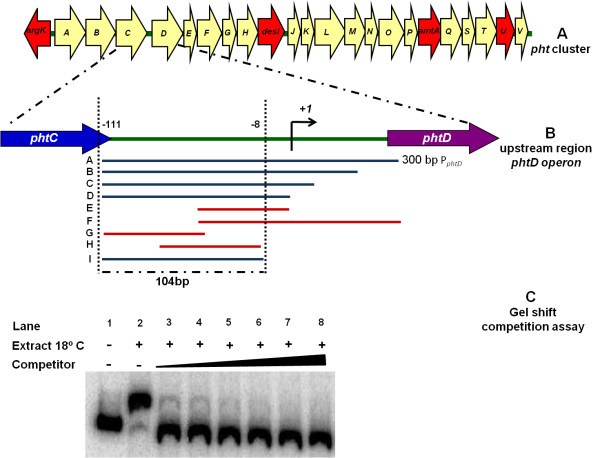
**Gel shift competition assays**. (A) Graphic representation of the *pht *region. Each arrow represents an individual gene, with the direction of the arrow indicating the direction of transcription. Red arrows indicate genes whose function have been previously reported (B) Detailed view of the *phtD *operon upstream region indicating the P_*phtD *_fragments used as unlabeled DNA competitors. The blue bars represent the probes able to compete the DNA-protein complex, while the red bars represent probes unable to compete the complex. The fragment "I" corresponding to the region of 104 bp defined as the binding site for protein. (C) An example of gel shift competition assays used in this case, fragment "I" as competitor. These assays were carried out using crude protein extracts of *P. syringae *pv. phaseolicola NPS3121 grown at 18°C in M9 minimal medium and increasing concentrations of different unlabeled DNA fragments indicated in (B) as competitors. We show the gel shift competition assay performed with the 104 bp probe, which was identified as the minimum region necessary to bind a putative transcription factor. The concentration of unlabeled DNA competitors was as follows: lanes 1 and 2, no competitor DNA; lane 3, 25 ng (0.36 pmol); lane 4, 50 ng (0.73 pmol); lane 5, 60 ng (0.87 pmol); lane 6, 100 ng (1.46 pmol); lane 7, 150 ng (2.18 pmol); and lane 8, 200 ng (2.9 pmol).

The *phtD *operon includes eight genes from *phtD *to *phtK*, whose expression can be driven either from the promoter upstream of *phtD*, or from read-through from the *phtA *promoter located upstream (Figure 1A). The transcription initiation site for the *phtD *operon was determined to be 127 bp upstream of the probable initiation codon, and analysis of this promoter region did not show any similarity with binding sites reported for bacterial sigma factors [10]. Therefore, in order to identify putative transcriptional regulators of the *phtD *operon, we performed mobility shift assays to analyze the presence of DNA-binding proteins in crude extracts of *P. syringae *pv. phaseolicola NPS3121. A 300 bp radiolabeled DNA fragment (P_*phtD*_), spanning positions -111 to +188 relative to the transcription start site of the *phtD *operon was used as probe (Figure 1B). Radiolabeled P_*phtD *_fragment was incubated with cellular protein extracts from *P. syringae *pv. phaseolicola NPS3121 grown at 28°C and 18°C under appropriate binding conditions. Mobility shift assays showed that the fragment was able to form a specific DNA-protein complex with a protein found in extracts of cells grown at 18°C (the optimal temperature for toxin production). Likewise, the same retarded mobility complex was obtained with extracts from cultures grown at 28°C, indicating that the presence of the interacting protein is independent of temperature (see Additional file 1).

To determine the specificity and localization of the observed protein-DNA complex, mobility shift assays were carried out using different P_*phtD *_fragments as unlabeled competitors (indicated in Figure 1B). These assays showed that the retarded band was effectively competed by the full-length probe (A) and by fragments B, C, D and I, thus indicating that the observed protein-DNA interaction is located in a 104 bp region that spans positions -111 to -8, relative to the *phtD *operon transcription start site (Figure 1B and 1C). Although shorter length probes (G, H) were used in gel shift competition assays, these were unable to compete the DNA-protein complex (data not shown). Incubation of crude cell extracts with specific or non-specific DNA probes also showed that the DNA-protein binding is specific and is localized in the above-mentioned position (data not shown). These results suggested that a putative transcription factor of the *phtD *operon is present in *P. syringae *pv. phaseolicola NPS3121 during growth at both temperatures.

### The putative transcription factor of the *phtD *operon is encoded outside of the Pht cluster

In general, genes that participate in the synthesis of phytotoxins are grouped together in a particular chromosomal region, within which are encoded both structural genes and regulatory proteins involved in the process [24]. However, in the case of *P. syringae *pv. phaseolicola it is unknown whether all genes necessary for the synthesis and regulation of phaseolotoxin are found within the Pht cluster. We performed a bioinformatic analysis for each of the predicted ORFs of the Pht cluster, in a search for DNA binding motifs using the Pfam database (http://pfam.sanger.ac.uk/) [25]. According to this analysis, no DNA binding motif was found in the Pht gene cluster (data not shown).

**Figure 2:**
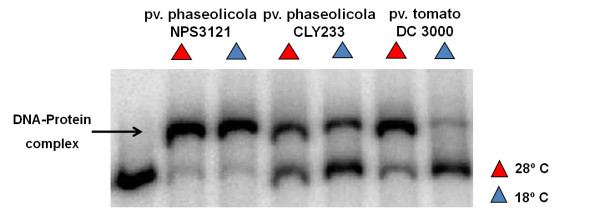
**Gel shift assays with crude extracts of different pathovars of *P. syringae***. Radiolabeled P_*phtD *_fragment was incubated with protein extracts of *P. syringae *pv. phaseolicola strains NPS3121and CLY233, and *P. syringae *pv. tomato DC3000, grown at 18°C and 28°C in M9 minimal medium. Gel shift assays were carried out under conditions similiar to those used with crude extracts of the wild-type strain. The arrow indicates the DNA-protein complex.

In order to assess whether the putative transcription factor of the *phtD *operon as revealed through the mobility shift analysis was encoded outside or within the Pht region, gel-shift assays were performed using crudes extracts from *P. syringae *pv. phaseolicola strain CLY233, a non-toxigenic strain lacking the Pht cluster and *P. syringae *pv. tomato DC3000 (non phaseolotoxin-producer) grown at 18°C and 28°C in M9 minimal medium. Incubation of the radiolabeled P_*phtD *_fragment with crude protein extracts of the above mentioned strains demonstrated the presence of a retarded mobility complex similar to that obtained with protein extracts of *P. syringae *pv. phaseolicola NPS3121 (Figure 2). Mobility shift competition assays with specific and non-specific probes indicated that the observed DNA-protein binding was specific for the P_*phtD *_region (data not shown). These results indicated that the putative transcription factor binding upstream of *phtD *was encoded by a gene located outside of Pht region that is shared with other pathovars and thus is not specific for phaseolotoxin synthesis, and also that its presence is independent of temperature. Therefore, these results suggest that upon transfer of the Pht cluster horizontally, the regulation of phaseolotoxin synthesis adapted to pre-existing regulatory mechanisms of *P. syringae *pv. phaseolicola NPS3121.

### A DNA sequence upstream of the *phtD *operon contains a putative binding site for the IHF protein

**Figure 3:**
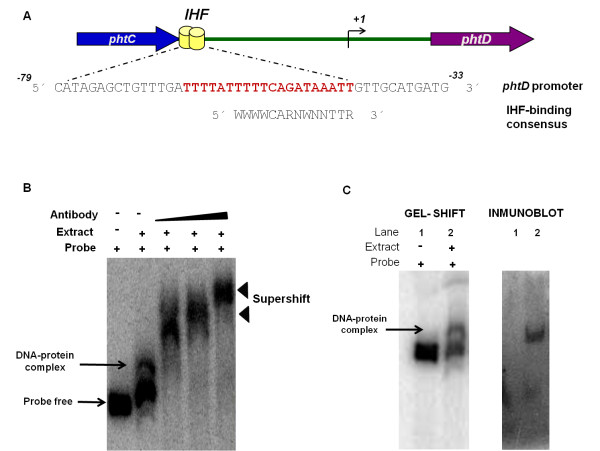
**Bioinformatic analysis of the sequence upstream of the *phtD *operon, and Supershift and Shift-western experiments to analyze the DNABII-family proteins binding activity to the P_*phtD *_fragment**. (A) Bioinformatic analyses. This panel schematizes the intergenic region between *phtC *and *phtD *where the IHF binding site position is represented with a yellow barrel. The alignment of the *phtD *IHF binding site with the consensus IHF binding site proposed by Kur *et al *[27] is also shown. The sequence identified as the putative IHF binding site in the *phtD *promoter is shown in bold red letters. *W*: A or T; *R*: A or G; *N*, any base. (B) Supershift assays. Analyses were conducted using increasing concentrations of anti DNAB-II family proteins antibody. Supershift signals were observed when antibody was added to the reaction mixture. The specific DNA-protein complex is indicated by a solid arrow. Supershift bands are indicated by solid arrowheads. (C) Shift-western experiment. Gel shift assays with the P_*phtD *_probe were performed as described in the Methods, followed by transfer of proteins onto nitrocellulose membranes, which were probed with antibody to DNA-binding proteins of DNAB-II family. To identify the signal, the images were analyzed using Quantity-one software (BIO-RAD) following the manufacturer's instructions. Panel I depicts a standard gel mobility assay with radiolabeled P_*phtD *_probe. Lane 1, free probe; lane 2, DNA-protein complex. Panel II: Immunoblot using polyclonal antibody. Lanes correspond to those of Panel I. The arrow indicates the position of the gel shift band.

Once the binding site for the putative *phtD *regulatory protein had been delimited to a 104 bp region and it was determined that this protein was encoded outside the Pht cluster, we evaluated the presence of putative *cis-*acting elements within the *phtD *promoter region using a transcription factor search program (BPROM, http://www.softberry.com) [26]. Sequence analysis revealed the presence of a potential binding site for the DNA-binding/bending protein IHF. This sequence was located at positions -64 to -44, relative to the start of *phtD *transcription, and showed similarity to the consensus IHF binding site proposed by Kur *et al. *[27] (Figure 3A).

### Members of the DNABII family (HU or IHF) interact with the P_*phtD *_fragment

IHF is a member of the DNABII DNA-binding protein family, which includes HU (a histone-like protein from *E. coli *strain U93) and IHF proteins [28]. The IHF protein has been reported to regulate the expression of several genes, some of which are involved in virulence factor synthesis [29,30]. To assess whether IHF might interact with the *phtD *promoter region, and whether it was involved in the formation of the complex observed in gel mobility shift assays, we performed supershift assays. Supershift assays were carried out using a polyclonal antibody directed against DNA-binding proteins of the DNABII family (IHF and HU proteins). The addition of the antibody generated a supershifted complex when compared with a negative control, where no antibody was added (Figure 3B). On the other hand, when the probe was incubated with the anti-DNAB-II antibody without protein extract, neither shifted nor supershifted band was observed, ruling out nonspecific antibody-probe interactions. Furthermore, no supershifted band was revealed when unrelated antibodies were evaluated, again validating the specificity of the antibody used (see Additional file 1). These assays indicated that members of the DNAB-II family (IHF or HU) are involved in the protein-DNA complex that forms at the *phtD *promoter region. Finally, to provide additional confirmation that IHF or HU contributed to the gel mobility shift results, we performed shift-western experiments, in which shifted bands were transferred to nitrocellulose membranes and incubated with anti-DNABII family protein antibodies. Incubation with antibodies yielded one band at a position identical to that of the shifted band (Figure 3C), supporting the presence of a DNAB-II family DNA-binding protein (IHF or HU) in the complex identified by gel mobility assays.

### IHF protein interacts with the *phtD *operon promoter region

**Figure 4:**
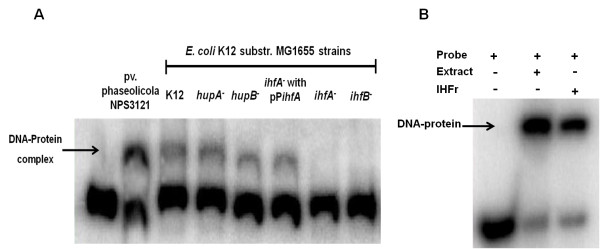
**Gel shift assays using *Escherichia coli *mutant strains and purified IHF protein**. Gel shift assays were performed as described in Methods. (A) Protein extracts of *E. coli *mutants for subunits of HU (*hupA, hupB*) and IHF proteins (*ihfA *and *ihfB*) were used in these assays. The arrow indicates the DNA-protein complex formed. (B) Gel shift assay using the purified IHF protein from *E. coli *(IHFr), which produces a retarded signal similar to that obtained with the extract of *P. syringae *pv. phaseolicola. The probe used in this assay corresponds to the 104 bp region. The band shifted is indicated by an arrow.

To determine the identity of the protein observed in gel shift assays, we analyzed crude protein extracts of *E. coli *single mutants having, deletions in the genes coding for the alpha and beta subunits of IHF and HU proteins by gel mobility shift assays. The bacterial strains were grown in LB at 37°C until the cells reached the early stationary phase, when IHF levels are reported to increase and even small amounts of HU protein are observed [31]. Incubation of the P_*phtD *_probe with crude extracts from *E. coli *strains K12 *wild type, hupA^-^, and hupB^-^*, showed a retardation signal similar to that obtained with extracts of *P. syringae *pv. phaseolicola NPS3121, indicating that mutations in genes encoding HU protein subunits have no effect on the presence of the putative *phtD *regulatory protein. However, when crude extracts of *E. coli *mutants *ihfA^- ^and ihfB^- ^*were assayed, no retarded signal was observed (Figure 4A). These results strongly suggest that the protein involved in the DNA-protein complex is IHF. To validate these results, two types of additional experiments were performed: 1) mobility shift competition assays using the *algD *promoter region and 2) mobility shift assays with a complemented *E. coli ihfA^- ^*strain.

Previously, it was reported that the promoter region of the *algD *gene in *P. aeruginosa *contains a functional binding site for the IHF protein [32]. This site has also been found in the promoter region of the orthologous gene in *P. syringae *pv. phaseolicola 1448A. For that reason, we decided to use the promoter region of the *algD *gene of 1448A, which contains a putative IHF binding site, as a competitor in gel shift assays. The results showed that the retarded mobility signal progressively decreased, compared to the DNA-protein complex, indicating that increasing concentrations of competitor DNA titrated the protein. However, when the promoter region of the same gene without the putative IHF binding site was used as a competitor, the retarded signal intensity was not altered (see Additional file 1). Additionally, a second experiment was conducted where the *P. syringae *pv. phaseolicola NPS3121 IHF alpha subunit gene was cloned in the pCR4-TOPO vector, creating the plasmid pP*ihfA*, which was then introduced into the *E. coli ihfA^- ^*mutant. Crude extracts of the complemented *E. coli *strain were used in mobility shift assays to analyze the binding activity of the P_*phtD *_fragment. Mobility shift assays showed the presence of a retarded signal similar to that obtained with our *P. syringae *pv. phaseolicola strain, indicating that the presence of the *ihfA *gene *in trans *is capable of restoring the formation of the DNA-protein complex (Figure 4A). Finally, strong evidence concerning the identity of the P_*phtD *_binding protein was obtained through gel shift assays using IHF protein purified from *E. coli*, which showed the presence of a retarded signal whose position was identical to that formed with the protein present in extracts of *P. syringae *pv. phaseolicola NPS3121 (Figure 4B). These results unambiguously demonstrate that the IHF protein interacts with the *phtD *promoter region and is probably involved in regulation of this operon.

### The IHF protein exerts a negative effect on the expression of the *phtD *operon in *E. coli*

**Figure 5:**
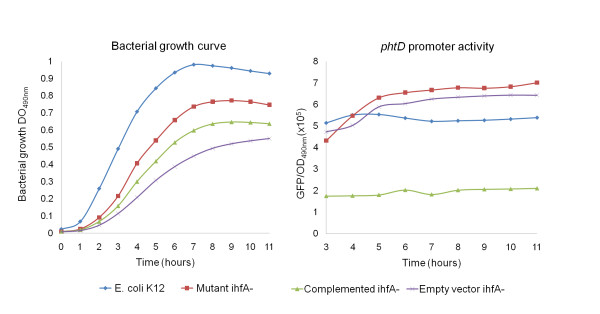
**Promoter activity of the *phtD *operon in *Escherichia coli *background**. (A) Growth curve of *E. coli *strains carrying the *phtD*::gfp transcriptional fusion grown in LB broth. (B) Fluorescence activity of *phtD*::gfp in the *E. coli *background.

To assess the participation of the IHF protein in regulating *phtD *operon expression, a transcriptional fusion of the *phtD *promoter was made to the *gfp *reporter gene creating the pJLAG plasmid with the intention of evaluating the expression from this construct in an IHF^- ^background of our *P. syringae *pv. phaseolicola strain. However, despite the fact that several strategies were attempted to obtain mutations in the subunits of the *P. syringae *pv. phaseolicola NPS3121 IHF protein, these mutants could not be obtained. Nevertheless, because the amino acid sequences of the *P. syringae *pv. phaseolicola IhfA and IhfB proteins are 86% and 73% identical to the *E. coli *IhfA and IhfB proteins respectively (data not shown), and since previous reports demonstrated that the *E. coli *IHF protein can functionally replace the IHF protein of some *Pseudomonas *and *viceversa *[33], we decided to perform the assays with the *ihfA*^- ^mutant strain of *E. coli*. We examined the expression of the *phtD::gfp *transcriptional fusion (pJLAG) in wild type *E. coli *K12 and *ihfA^- ^*mutant backgrounds. The expression of *phtD::gfp *was increased in the *ihfA^- ^*background, in comparison to the expression observed in the wild type *E. coli *K12 strain. On other hand, when the expression of the *phtD::gfp *transcriptional fusion was examined in the *ihfA^- ^*mutant complemented with the *ihfA *gene of *P. syringae *pv. phaseolicola NPS3121, we observed a clear reduction in fluorescence levels, suggesting a decrease in gene expression (Figure 5). However, to investigate the possibility that the decrease in *phtD *promoter expression was related to the decrease in growth rate observed in this strain, possibly due to over-expression of the *ihfA *gene, we evaluated the expression of the *phtD::gfp *fusion in the *ihfA^- ^*mutant transformed with the PCR 4-TOPO vector (without *ihfA *gene). The results of these experiments showed that the decrease in the growth rate was possibly due to the presence of an additional plasmid and not to the presence of the *ihfA *gene, which excludes a possible toxic effect. Likewise, the results showed that the decrease in the expression observed from the *phtD::gfp *fusion in the complemented *ihfA^- ^*mutant was, due solely to the presence of the *ihfA *gene *in trans*, and not to the observed decrease in growth (Figure 5). These results indicate that the IHF protein negatively regulates expression of the *phtD *operon in *E. coli*.

### Mutations in the putative IHF binding site affect the DNA-protein interaction

**Figure 6:**
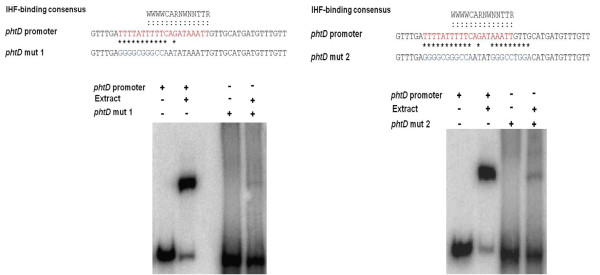
**Evaluation of the effect of mutations in the proposed IHF binding site**. Gel mobility shift assays using the mutant probes of fragment I (104 bp). Panel A shows the assays using mutant probe 1, which contains changes in the dA-dT rich upstream region as well as changes of C to A and G to T in the consensus sequence. These changes caused a decrease of 89% with respect to the control. Panel B shows assays using mutant probe 2, which also includes mutations in the TTR region of the consensus sequence, causing an 86% decrease in the retarded signal. The asterisks indicate the bases modified. The bold red letters indicate the proposed site for IHF binding.

Since the IHF site found in the *phtD *operon promoter region has 83% similarity with the reported consensus sequence, we evaluated the role of this sequence on the DNA-protein interaction. To this end, 104 bp synthetic oligonucleotides corresponding to the minimum binding region for IHF were designed with mutations at bases previously reported to be necessary for IHF protein binding. The selected mutations were based upon those previously shown to severely affect IHF binding [34]. Two mutant probes were analyzed. Mutant probe 1 (L100271-L100272) has changes in the dA-dT rich upstream region as well as changes of C to A and G to T of the consensus sequence. Gel mobility shift assays with mutant probe 1 clearly show a dramatic decrease in the amount of retarded signal (89%) as compared to the amount of signal obtained with the wild type probe (Figure 6A). These results indicate that the changes introduced in this probe decrease the P_*phtD*_-IHF interaction. Likewise, gel mobility shift assays with mutant probe 2 (L100275-L100276), which also includes mutations in the TTR region of the consensus sequence, showed a clear decrease in the retarded signal as compared to the control (86%) (Figure 6B). These results demonstrate the role of this sequence in IHF protein binding.

## Discussion

Phaseolotoxin is an important virulence factor of *P. syringae *pv. phaseolicola, whose synthesis involves genes in the Pht cluster. The expression of these genes is higher at 18°C than at 28°C, which is consistent with conditions of phaseolotoxin synthesis [10]. So far, the regulatory mechanism involved in the production of this phytotoxin has not been elucidated, and the only known fact is the effect of low temperatures on its synthesis [7]. In the present work we initiated study of the regulatory pathway involved in phaseolotoxin synthesis in *P. syringae *pv. phaseolicola NPS3121 by focusing on the control of *phtD *operon expression. In this study we report the binding of the IHF protein to the *phtD *promoter region and a possible role for this protein in controlling the expression of this operon.

Mobility shift assays using the region upstream of the *phtD *operon as a probe showed the formation of a DNA-protein complex that clearly indicates the presence of a binding site for a regulatory protein within this region. These data also indicate that the presence of this protein is independent of temperature, as it was found in crude extracts obtained at both 28°C and 18°C. The minimal region necessary for the binding of this protein was defined by competition assays to be a region of 104 bp, a size greater than that reported for most DNA-binding proteins, which are typically 20-40 bp [35]. This result suggests that the DNA-protein interaction observed in *phtD *not only depends on the recognition of specific sequences but also depends on specific DNA structures that can only form in the 104 bp fragment. A similar requirement has been reported for some regulatory proteins, such as H-NS, which requires a curved DNA structure for its binding [36-38].

The assays with *P. syringae *pv. phaseolicola strain CLY233 (which lacks the Pht cluster) and *P. syringae *pv. tomato DC3000 (non phaseolotoxin-producer), show a retardation signal identical to that obtained with our working strain, indicating that the protein binding to the *phtD *promoter region is encoded outside of the Pht cluster and is not exclusive to the phaseolicola pathovar, given its presence in other pathovars, futher suggesting that this protein could be a global regulator. These results validate the bioinformatic prediction that did not reveal a DNA-binding motif for any predicted Pht cluster ORF, suggesting that none of these proteins encode a DNA-binding protein, although evidence in some cases suggests a regulatory role for the product of some genes found within the Pht cluster [10]. Our findings resemble previous reports in *P. syringae *pv. syringae, in which the *syr-syp *gene clusters involved in syringomycin *(syr) *and syringopeptin *(syp) *synthesis, are regulated by the SalA and GacS/GacA proteins, which are localized outside of this region and also present in other pathovars. However, the regulation of these phytotoxins also depends on regulatory proteins present in the *syr-syp *region [24].

An approximation for the identity of the *phtD *binding protein was obtained by supershift and shift-western assays, which indicated that DNA-binding proteins of the DNABII family (HU or IHF) are involved in the formation of the protein-DNA complex observed in the *phtD *promoter region. These results are consistent with the bioinformatic analysis, which revealed the presence of a potential binding site for the IHF protein, at position -64 to -44, relative to the start of *phtD *transcription.

Finally, the identity of the *phtD *binding protein was determined by mobility shift assays using *E. coli *strains mutated in each of the genes encoding subunits of HU and IHF proteins. The absence of retardation signal in *ihfA^- ^*and *ihfB^- ^*mutants clearly indicates a role for these proteins in the formation of the DNA-protein complex, thus demonstrating that IHF protein binds to the *phtD *promoter region. Further evidence for the binding of IHF to this region was provided by cell extracts from a complemented *E. coli ihfA^- ^*strain, in which the retarded signal was restored by the presence of the *P. syringae *pv. phaseolicola *ihfA *gene acting *in trans*. Finally, mobility shift assays using purified IHF protein confirmed that the protein binding the *phtD *promoter region was IHF.

IHF is a small basic DNA-binding protein conserved in Gram-negative bacteria that belongs to the class of so-called nucleoid associated proteins (NAP's) [39,40]. The IHF protein consists of two heterologous subunits, IHFα and IHFβ which are encoded in different transcription units by the homologous *ihfA (himA) *and *ihfB (himD) *genes, respectively. Both subunits also share significant homology with the subunits of the HU protein, a nonspecific DNA binding protein that also belongs to the same protein family. Unlike the HU protein, the IHF protein recognizes a specific consensus sequence: WCARNWNNTTR (where W represents A or T and R represents A or G), which introduces a bend of 180° into the DNA, centered at the 5'end of the 5'-WWWCAR-3' element in the binding site [35,39]. In addition, 5'-proximal bases with high dA-dT content, are also thought to be required for binding of this protein at some sites [34,41]. The IHF-binding sequence predicted upstream of the *phtD *region is 83% identical to the reported consensus sequence, and only two changes (A to T; T to A, or G) are observed in the 3'-end. In addition, an A-T rich region is found upstream of this sequence, strongly suggesting a role for these sequences in the binding of the IHF protein. Mobility shift assays with mutant probes clearly demonstrated a role for these residues in the P_*phtD*_-IHF interaction. Similarly, our proposal for the requirement of a change in the DNA structure for IHF binding to the *phtD *operon is somewhat supported by various reports which demonstrate that besides the interaction with consensus sequences, the IHF protein requires a curved DNA structure for binding [38].

The IHF protein contributes in an important way to the function of a wide variety of macromolecular processes in bacteria and is recognized as a global regulation factor in the transcription of many genes. IHF can alter gene expression in a number of ways, including positive and negative effects on transcription, and its role as a regulator of virulence gene expression has increasingly been determined [39,42]. The role of IHF protein in regulating *phtD *operon expression was examined through the analysis of a *phtD::gfp *transcriptional fusion in an *E. coli *K12 *ihfA^- ^*mutant background, which clearly showed higher transcriptional activity than that observed in the wild type background. This activity significantly decreases when the *ihfA^- ^*mutant strain is complemented *in trans *with the *ihfA *gene of *P. syringae *pv. phaseolicola NPS3121, suggesting that the IHF protein has a negative effect on the expression of the *phtD *operon in *E. coli*. Because some reports have demonstrated that the *E. coli *IHF protein can functionally replace IHF proteins of some *Pseudomonas *species, and since this protein is not modulated by interactions with inducer or co-repressor molecules, as are most transcription factors [33,35], we propose that the IHF protein also exerts a negative effect on *P. syringae *pv. phaseolicola NPS3121 *phtD *operon expression.

IHF has been shown to act as a negative regulator through several mechanisms. In some cases, IHF seems to act as a classical repressor by binding to DNA within the RNA polymerase recognition site and excluding the polymerase from the promoter. IHF may also act indirectly as a repressor, collaborating with a gene-specific repressor or obstructing the binding of an activator. Alternatively, IHF can repress transcription in concert with other nucleoid proteins and global or gene-specific transcriptional regulators to create a higher-order nucleoprotein complex that forms an inhibitory promoter architecture [35,37,42]. The way in which IHF could act to repress the *phtD *operon is unknown, although according to the position of the predicted IHF binding site (-64 to -44), its role as a classical repressor may be dismissed. The fact that the expression of *phtD::gfp *transcriptional fusion is not completely depressed, despite the presence of IHF on a multiple copy plasmid, suggests the participation of other regulatory proteins with which the IHF protein may interact to regulate *phtD *operon expression.

On other hand, a common mechanism for transcriptional regulation of *phtD *and *phtM*, due to the presence of conserved regions in promoters of these genes has been suggested [10], however the bioinformatic analysis did not reveal IHF binding sites in the *phtM *promoter region. In addition, mobility shift competition assays showed that this region is unable to compete the retarded signal in *phtD*, indicating that the IHF protein does not bind to the upstream region of *phtM *(data not shown).

Several lines of evidence have postulated that the genes of the Pht cluster form a genomic island (GI), which was acquired by horizontal gene transfer from a Gram positive bacterium [18-20]. Based on our findings, we propose that the regulation of this gene cluster (Pht cluster), became integrated into the global regulatory mechanism of the host-bacterium *P. syringae *pv. phaseolicola NPS3121, after the horizontal transfer event. This phenomenon of foreign DNA integration into the regulatory pathway of the host cell has been reported in other organisms and several examples of horizontally acquired genes that are regulated by global proteins exist in the literature. In Salmonella, genes within the SPI-1 pathogenicity island, which is thought to have originated outside the enteric bacteria, are positively regulated by the nucleoid protein Fis. Similarly, the virulence regulon in *Vibrio cholerae*, which is a mosaic of ancestral and horizontally acquired genes, uses the H-NS global regulator as a transcriptional repressor; as does enteropathogenic *E. coli*, where the H-NS protein represses the virulence genes in the LEE pathogenicity island (PAI) [43,44]. The role of the IHF protein in the regulation of transferred genes has also been reported, with this protein positively regulating the virulence genes TCP *(Toxin-Coregulated Pilus) *and CT (C*holera Toxin*) in *V. cholerae*, alleviating H-NS repression. Similarly the IHF protein directly activates the expression of genes in the LEE PAI in enteropathogenic *E. coli *[30,45]. It seems that the integration of foreign DNA into the global regulatory mechanisms of host bacterium is not unusual. Some authors suggest that this event allows the host cells to control the expression of transferred genes thus avoiding unregulated expression that could have harmful consequences besides having a high energy cost [46,47]. Based on our results, we suggest that in *P. syringae *pv. phaseolicola NPS3121, the control of some genes of the Pht cluster is dependent on the IHF protein.

## Conclusions

In this study we demonstrated that the regulatory protein IHF binds to the promoter region of the *phtD *operon, most likely exerting a negative control on expression of this operon. This is the first regulatory protein reported as part of the regulation system of phaseolotoxin synthesis in *P. syringae *pv. phaseolicola NPS3121, which suggests that regulation of gene expression within the Pht cluster has integrated into the global regulatory mechanisms. However, it is still necessary to dissect in detail the regulatory mechanism of the IHF protein and identify other regulators that will enable us to elucidate the regulatory pathway for phaseolotoxin production in *P. syringae *pv. phaseolicola NPS3121.

## Methods

### Bacterial strains, media and growth conditions

The bacterial strains and plasmids used in this study are listed in Additional file 2, Table S1. *P. syringae *strains: pv. phaseolicola NPS3121, pv. phaseolicola CLY233 and pv. tomato DC3000 were grown on M9 minimal medium at 18°C or 28°C. Pre-inoculums (25 ml) of *P. syringae *strains were grown overnight at 28°C in M9 medium with glucose (0.8%) as the carbon source. The cells were inoculated into 50 ml M9 minimal medium at OD_600 nm _0.1 and the cultures were incubated at 18°C and 28°C until they reached the transition phase (OD_600 nm _1.0). *Escherichia coli *wild type and mutant derivative strains, were routinely grown on Luria-Bertani (LB) medium at 37°C. When required, the following antibiotics were added: carbenicillin 100 μg μl^-1^, kanamycin 50 μg μl^-1^, rifampin 50 μg μl^-1^.

### Molecular biology techniques

Routine techniques were performed using standard protocols [48]. Genomic DNA of *P. syringae *pv. phaseolicola NPS3121 was isolated as described previously [49]. Plasmid DNA was isolated from *E. coli *using the QIAGEN^®^: plasmid midi kit following the manufacturer's instructions. PCR products were amplified with High Fidelity DNA Polymerase and Platinum supermix (Invitrogen, California USA) and purified with the QIAquick^® ^gel extraction kit (QIAGEN). Restriction enzymes were used according to *manufacturer's instructions*. Primers were designed using Vector NTI Software (Invitrogen, California USA) with reference to the previously reported Pht cluster sequence (Gen Bank DQ141263) [10]. The oligonucleotide primers used in this study are listed in Additional file 2, Table S2.

### Gel mobility shift assays

The probes used in gel shift assays were obtained by PCR amplification using the oligonucleotide pairs shown in Additional file 2. The double-stranded probes were end-labeled with ( ^32^P)-ATP using T4 polynucleotide kinase enzyme (Invitrogen, California USA). Gel shift assays were performed as previously described, with some modifications [50]. Briefly, protein extracts were prepared from *P. syringae *pv. phaseolicola NPS3121 grown in M9 minimal medium at 18°C and 28°C until reaching the transition phase (OD_600 nm _of 1.0). Cultures were centrifuged and the pellet was rinsed once with 1/20 volume of cold extraction buffer (25 mM Tris-HCl pH 8.0, 0.1 mM EDTA, 1 mM DTT, 10% glycerol and 0.04 mM PMSF), the cell pellet was freeze-thawed once, diluted in 1/20 volume in extraction buffer, and sonicated (3 times for 15s with intervals of 15s) in an ice bath using a Virsonic 60 sonicator (Virtis Company Inc). The cellular debris was pelleted by centrifugation at 13,000 r.p.m in a microcentrifuge, for 5 min at 4°C and discarded. Total protein was measured using the Bradford method with a BSA standard curve as control [51]. The binding reactions contained approximately 10 ng of the probe (0.051 pmol for P_*phtD *_and 0.146 pmol for fragment I), 30 μg of the appropriate protein extract, 0.5-1 μg poly(dI-dC), and 0.2 μg sonicated salmon sperm DNA, in a 20 μl total volume of binding buffer (25 mM Tris pH 7.5, 50 mM KCl, 1 mM EDTA, 1 mM DTT, 5% glycerol) and were incubated for 30 min at room temperature. Protein-DNA complexes were separated from unbound probe on 6.5% native polyacrylamide gels at 6 mA for 3-4 hrs, in 0.5X TBE buffer. Gels were vacuum-dried and exposed to a Phosphor screen (Molecular Dynamics). The image was captured by scanning on a STORM 860 (Molecular Dynamics) and analyzed with Quantity One software (BIO-RAD). To determine the specificity of the DNA-protein complexes observed, competition assays were carried out using increasing concentrations of specific and non-specific competitor DNA. A 300 bp-*Pvu*II fragment of pUC19 plasmid was used as non-specific competitor. To determine the localization of the DNA-protein complex, competition assays were performed with an excess of unlabelled wild-type probes, listed in Additional file 2, Table S3. When crude extracts of *P. syringae *pv. tomato DC3000 and *P. syringae *pv. phaseolicola CLY233 were assayed, the same gel shift assay conditions were used. For analysis of *E. coli *mutants, strains were grown at 37°C on LB broth until reaching an optical density of 1.2 (OD _600 nm_), and the conditions of the gel-shift assays were similar to those described above.

### Gel Mobility shift assays with purified IHF protein

Gel shift assays were performed essentially as described above with some changes. Purified IHF protein from *E. coli *(a generous gift from Dr. Steven Goodman) was used in these assays at a concentration of 2 μM. The probes used corresponded to the P_*phtD *_fragment (300 bp) (data not shown) and the fragment I (104 bp) obtained by PCR amplification. The probe concentration of the 104 bp used was 0.146 pmol. Protein-DNA complexes were separated from unbound probe on 8% native polyacrylamide gels under conditions previously mentioned.

### Electrophoretic mobility supershift assays

The antibody used in supershift assays is a polyclonal antibody that was raised in rabbit against DNA-binding proteins of the DNAB-II family (e.g. HU, IHF) (a generous gift from Dr. Steven Goodman). Prior to the addition of the radiolabeled probe, the protein extract was incubated with increasing concentrations of antibody for 20 min at room temperature. The probe was then added and the reaction continued for another 30 min at room temperature. Each reaction mixture was analyzed by gel shift assays as described above. In these assays only crude extracts of *P. syringae *pv. phaseolicola NPS3121 grown at 18°C were used.

### Shift–Western assays

The Demczuk method [52] was used to identify the protein components of the gel-shift assays in combination with the immunoblotting technique, with some modifications. Gel shift assays were carried out under the conditions mentioned above. Only crude extracts of the wild type strain grown at 18°C were evaluated, and the P_*phtD *_fragment was used as probe. The binding reactions were prepared in duplicate and subjected to electrophoresis. After completion of the gel shift assay, the gel was divided into two parts; one was exposed and used as control, while the other was blotted onto a nitrocellulose membrane at room temperature for 45 min at 20 V in a buffer containing 25 mM Tris pH 8.0, 192 mM Glycine and 5% methanol using a semidry blotting apparatus (Trans-blot SD, BIO-RAD). For immunoreactive detection, the membranes were first blocked overnight at 4°C in TBS containing 5% skimmed milk, and subsequent manipulations were done in the absence of skimmed milk. Primary antibody was applied at a dilution of 1:1000 and enhanced chemiluminescence protein detection was done using Amersham anti-rabbit peroxidase-conjugated antibodies as described by the manufacturer (Amersham Biosciences). To identify the signal, the images were overlapped using Quantity-one software (BIO-RAD) following the manufacturer's instructions.

### Complementation of *ihfA^- ^E. coli *mutant with the alpha-subunit gene of *P. syringae *pv phaseolicola NPS3121

Using the sequence of the 1448A strain (Gene Bank accession no. CP000058) [53], we designed primers to amplify the *ihfA *gene of *P. syringae *pv. phaseolicola NPS3121. The *ihfA *gene was obtained by PCR amplification using oligonucleotides L100258-L100259 (Additional file 2, Table S2), and cloned into the pCR4-TOPO vector, under control of the *lac*Z promoter (pP*ihfA*). The construct was mobilized into the *ihfA^- ^E. coli *K12 mutant via electroporation. The orientation of the construct was determined by restriction enzyme digestion. The induction of the gene was carried out with 1 mM isopropyl-β-D-thiogalactopyranoside (IPTG).

### Construction of a *phtD:gfp *transcriptional fusion

The plasmid pUA66, which contains the *gfpmut2 *reporter gene with a strong ribosome binding site, was used to construct a transcriptional fusion. A 416-bp fragment, corresponding to the intergenic region of *phtC-phtD *(-179 to +236) was obtained by PCR using primers L100269 phtD*Xho*I and L100270phtD*Bam*HI, which include suitable restriction sites (Additional file 2, Table S2). This region (416 bp) was previously delimited as the minimum required for differential expression of the *phtD *operon, in response to temperature changes (unpublished data). The amplicon was cloned into the *Xho*I-*Bam*HI sites of pUA66 to create pJLAG and orientation was validated by PCR. To evaluate the activity of the *gfp *reporter gene, constructs were mobilized into *E. coli *K12 and the *ihfA^- ^*mutant derivative of *E. coli *K12, by thermal shock.

### Measurement of promoter activity by fluorescence

Pre-inoculums (2 ml) of *E. coli *K12 strain and mutant *ihfA^- ^*strain carrying the *gfp *fusion were grown for 16 hours at 37°C with agitation in LB broth supplemented with kanamycin (50 μg/μl). The cultures were diluted 1:100 in LB broth with kanamycin to a final volume of 150 μl per well in flat-bottomed 96-well plates. Cultures were grown at 37°C with constant shaking and monitored in a Wallac Victor 3X multiwell fluorimeter. The parameters for measurements of growth and fluorescence were: fluorescence readings (filters F485, F535, 0.5s, CW lamp energy 10,000) and absorbance (OD) measurements (490 nm, P490, 0.5s). The time between repeated measurements was 1 hour. Promoter activity was determined as the ratio of fluorescence and optical density (GFP/OD_490 nm_).

### Evaluation of the effect of mutations in the proposed IHF binding site

Gel mobility shift assays were carried out under the conditions mentioned above using 8% native polyacrylamide gels to separate complexes. Only crude extracts of the wild type strain grown at 18°C were evaluated. The probes used in these assays are derived from annealed oligonucleotides, which were designed with mutations at bases corresponding to the putative IHF binding site. The sequences of these oligonucleotides are shown in additional file 2 (Table S4). For the preparation of ^32^P-labeled oligonucleotide probes, forward primers (L100271 and L100275) were end-labeled with ( ^32^P)-ATP using T4 polynucleotide kinase enzyme (Invitrogen, California USA), and unincorporated nucleotides were removed using the QIAquick Nucleotide removal kit (QIAGEN) following the manufacturer's instructions. Equimolar amounts of complementary oligonucleotides (L100271-L100272 and L100275-L100276 respectively) were mixed and annealed in annealing buffer (0.1 M NaCl, 10 mM Tris-HCl pH8.0,1 mM EDTA) at 100°C for 10 min and allowed to slowly cool to room temperature. The efficiency of the annealing was validated on 8% polyacrylamide gels (data not shown). As a control, we performed gel shift assays using the 104 bp wild type probe (without changes). Quantification of signal intensity was carried out using Quantity One software (BIO-RAD) following the manufacturer's instructions.

## Authors' contributions

JL A-G contributed to experimental design, performed experiments, analyzed the data and drafted the manuscript. A H-M participated in the experimental design and performed the construction and analysis of the transcriptional fusion. G P-P participated in the design of the study. L GB participated in the design of the study. A A-M conceived the study, contributed to experimental design, revised the data obtained, and edited the manuscript. All the authors read and approved the final manuscript.

## Supplementary Material

Additional file 1 **In this Power Point file we show the results of gel shift assays with the protein extracts of *P. syringae *pv. phaseolicola NPS3121 grown at 28°C and 18°C, as well as the supershift assays using unrelated antibodies, including anti-His, anti-GST, and anti Rlk**. Furthermore, we show the gel competition assays using the *algD *promoter as competitor. Detection of binding to P_*phtD *_in extracts of *P. syringae *pv. phaseolicola NPS3121. Gel shift assays was performed using a radiolabeled P_*phtD *_fragment (-111 to +188) and crude extracts of *P. syringae *pv. phaseolicola NPS3121 grown at 18°C and 28°C in M9 minimal medium. Probe concentration was 0.05 pmol and protein concentration of crude extracts in each reaction was as follows: lane 1, no protein; lanes 2 and 3, 30 g. DNA-protein complex is indicated by an arrow. Supershift assays using unrelated antibodies. The assays were carried out using unrelated antibodies, including anti-His, anti-GST (both commercially available), and anti-Rlk, which validated the specificity of the anti-DNABII antibody. Furthermore, we show control experiments in which the DNA probe was mixed with the DNA-BII antibody in the absence of protein extract. The retarded and super-retarded complexes are indicated by an arrow. Gel shift competition assays with the *algD *promoter. Panel A shows the competition assays using the *algD *promoter region (500 bp), which includes the IHF binding site reported by Wozniak [32] as competitor. Competitors were added in increasing concentrations: 50 ng (0.15 pmol), 60 ng (0.18 pmol), 100 ng (0.3 pmol), 150 ng (0.45 pmol), 200 ng (0.6 pmol), and 300 ng (0.9 pmol). Panel B depicts the competition assays with the *algD *promoter region (265 bp) that does not contain the IHF binding site. The competitor concentration used was: 50 ng (0.29 pmol), 60 ng (0.34 pmol), 100 ng (0.57 pmol), 150 ng (0.86 pmol), 200 ng (1.14 pmol), and 300 ng (1.72 pmol). Click here for file

Additional file 2 **This Word file contains tables listing the strains and plasmids used in this study, as well as the sequence of oligonucleotides and probes used in gel shift assays**. Click here for file

## References

[B1] Mitchell RE Bean halo-blight toxin Nature 1976 260 75 76 10.1038/260075a0

[B2] Mitchell RE Isolation and structure of a chlorosis inducing toxin of *Pseudomonas phaseolicola* Phytochemistry 1976 15 1941 1947 10.1016/S0031-9422(00)88851-2

[B3] Mitchell RE Bieleski RL Involvement of phaseolotoxin in Halo blight of beans Plant Physiol 1977 60 723 729 10.1104/pp.60.5.723 16660172 PMC542702

[B4] Templeton MD Sullivan PA Shepherd MG The inhibition of ornithine transcarbamoylase from *Escherichia coli *W by phaseolotoxin Biochem J 1984 224 379 388 6393952 10.1042/bj2240379 PMC1144443

[B5] Ferguson AR Johnston JS Phaseolotoxin: chlorosis, ornithine accumulation and inhibition of ornithine carbamoyltransferase in different plants Physiol Plant Pathol 1980 16 269 275 10.1016/0048-4059(80)90041-7

[B6] Goss RW The relation of temperature to common and halo blight of beans Phytopathology 1970 30 258 264

[B7] Nüske J Fritsche W Phaseolotoxin production by *Pseudomonas syringae *pv. phaseolicola: the influence of temperature J Basic Microbiol 1989 29 441 447 10.1002/jobm.3620290713 2600779

[B8] Hernández-Morales A De la Torre-Zavala S Ibarra-Laclette E Hernández-Flores JL Jofre-Garfias AE Martínez-Antonio A Álvarez Morales A Transcriptional profile of *Pseudomonas syringae *pv phaseolicola NPS3121 in response to tissue extracts from a susceptible *Phaseolus vulgaris *L. cultivar BMC Microbiology 2009 9 257 10.1186/1471-2180-9-257 20003402 PMC2803797

[B9] Reuter G Enzymatic regulation of microbial phytoeffector biosynthesis Progress in industrial microbiology 1989 27 271 281

[B10] Aguilera S López-López K Nieto Y Garcidueñas-Piña R Hernández-Guzmán G Hernández-Flores JL Murillo J Álvarez-Morales A Functional characterization of the gene cluster from *Pseudomonas syringae *pv. phaseolicola NPS3121 involved in synthesis of phaseolotoxin J Bacteriol 2007 189 2834 2843 10.1128/JB.01845-06 17237165 PMC1855804

[B11] Peet RC Panopoulos NJ Ornithine carbamoyltranferase and phaseolotoxin immunity in *Pseudomonas syringae *pv. phaseolicola EMBO J 1987 6 3585 3591 16453811 10.1002/j.1460-2075.1987.tb02689.x PMC553825

[B12] Mosqueda G Van de Broeck G Saucedo O Bailey AM Álvarez-Morales A Herrera-Estrella L Isolation and characterization of the gene from *Pseudomonas syringae *pv. phaseolicola encoding the phaseolotoxin-insensitive ornithine carbamoyltransferase Mol Gen Genet 1990 222 461 466 10.1007/BF00633857 2274044

[B13] Hatziloukas E Panopoulos NJ Delis S Prosen DE Schaad NW An open reading frame in the approximately 28-kb *tox-argk *gene cluster encodes a polypeptide with homology to fatty acid desaturases Gene 1995 166 83 87 10.1016/0378-1119(95)00569-5 8529898

[B14] Hernández-Guzmán G Álvarez-Morales A Isolation and characterization of the gene coding for the amidinotransferase involved in the biosynthesis of phaseolotoxin in *Pseudomonas syringae *pv. phaseolicola Mol Plant-Microbe Interact 2001 14 545 554 10.1094/MPMI.2001.14.4.545 11310742

[B15] Arai T Kino K A novel L-amino acid ligase is encoded by a gene in the phaseolotoxin biosynthetic gene cluster from *Pseudomonas syringae *pv phaseolicola 1448A Biosci Biotechnol Biochem 2008 72 3048 3050 10.1271/bbb.80439 18997422

[B16] Tamura K Imamura M Yoneyama K Kohno Y Takikawa Y Yamaguchi I Takahashi H Role of phaseolotoxin production by *Pseudomonas syringae *pv. *actinidae *in the formation of halo lesions of kiwifruit canker disease Physiol Mol Plant Pathol 2002 60 207 214 10.1006/pmpp.2002.0405

[B17] Tourte C Manceau C A strain of *Pseudomonas syringae *which does not belong to pathovar *phaseolicola *produces phaseolotoxin European J Plant Pathol 1995 101 483 490 10.1007/BF01874471

[B18] Sawada H Suzuki F Matsuda I Saitou N Phylogenetic analysis of *Pseudomonas syringae *pathovars suggests the horizontal gene transfer of *argK *and the evolutionary stability of *hrp *gene cluster J Mol Evol 1999 49 627 644 10.1007/PL00006584 10552044

[B19] Sawada H Kanaya S Tsuda M Suzuki F Azegami K Saitou N A phylogenomic study of the OCTase genes in *Pseudomonas syringae *pathovars: The horizontal transfer of the *argK*-tox cluster and the evolutionary history of OCTase genes on their genomes J Mol Evol 2002 54 437 457 10.1007/s00239-001-0032-y 11956683

[B20] Genka H Baba T Tsuda M Kanaya S Mori H Yoshida T Noguchi MT Tsuchiya K Sawada H Comparative analysis of *argK-tox *clusters and their flanking regions in phaseolotoxin-producing *Pseudomonas syringae *pathovars J Mol Evol 2006 63 401 414 10.1007/s00239-005-0271-4 16927007

[B21] Zhou D Yang R Global analysis of gene transcription regulation in prokaryotes Cell Mol Life Sci 2006 63 2260 2290 10.1007/s00018-006-6184-6 16927028 PMC11136394

[B22] Browning DF Busby SJW The regulation of bacterial transcription initiation Nat Rev Microbiol 2004 2 1 9 10.1038/nrmicro787 15035009

[B23] Rowley KB Xu R Patil SS Molecular analysis of thermoregulation of phaseolotoxin-resistant ornithine carbamoyltransferase (argK) from *Pseudomonas syringae *pv. phaseolicola Mol Plant-Microbe Interact 2000 13 1071 1080 10.1094/MPMI.2000.13.10.1071 11043468

[B24] Bender CL Alarcón-Chaidez F Gross DC *Pseudomonas syringae *Phytotoxins: Mode of action, regulation and biosynthesis by peptide and polyketide synthetases Microbiol Mol Biol Rev 1999 63 266 292 10357851 10.1128/mmbr.63.2.266-292.1999 PMC98966

[B25] Pfam http://pfam.sanger.ac.uk/

[B26] BPROM http://www.softberry.com

[B27] Kur J Hasan N Szybalski W Physical and biological consequences of interactions between integration host factor (IHF) and coliphage lambda P'_R _promoter and its mutants Gene 1989 81 1 15 10.1016/0378-1119(89)90331-4 2553535

[B28] Swinger KK Rice PA IHF and HU flexible architects of bent DNA Curr Opin Struct Biol 2004 14 28 35 10.1016/j.sbi.2003.12.003 15102446

[B29] Sieira R Comerci DJ Pietrasanta LI Ugalde RA Integration host factor is involved in transcriptional regulation of the *Brucella abortus virB *operon Mol Microbiol 2004 54 808 822 10.1111/j.1365-2958.2004.04316.x 15491369

[B30] Stonehouse E Kovacikova G Taylor RK Skorupski K Integration host factor positively regulates virulence gene expression in *Vibrio cholera* J Bacteriol 2008 190 4736 4748 10.1128/JB.00089-08 18456804 PMC2446820

[B31] Azam TA Iwata a Nishimura A Ueda S Ishihama A Growth phase-dependent variation in protein composition of *Escherichia coli *nucleoid J Bacteriol 1999 181 6361 6370 10515926 10.1128/jb.181.20.6361-6370.1999 PMC103771

[B32] Wozniak DJ Integration host factor and sequences downstream of the *Pseudomonas aeruginosa algD *transcription start site are required for expression J Bacteriol 1994 176 5068 5076 8051019 10.1128/jb.176.16.5068-5076.1994 PMC196346

[B33] Calb R Davidovitch A Koby S Giladi H Goldenberg D Margalit H Holtel A Timmis K Sánchez-Romero JM De Lorenzo V Oppenheim AB Structure and function of the *Pseudomonas putida *integration host factor J Bacteriol 1996 178 6319 6326 8892836 10.1128/jb.178.21.6319-6326.1996 PMC178507

[B34] Hales LM Gumport RI Gardner JF Determining the DNA sequence elements required for binding integration host factor to two different target sites J Bacteriol 1994 176 2999 3006 8188600 10.1128/jb.176.10.2999-3006.1994 PMC205457

[B35] Wagner R Regulation by transcription factors Transcription regulation in prokaryotes 2000 Oxford Press 193 260

[B36] Schröder O Wagner R The bacterial regulatory protein H-NS a versatile modulator of nucleic acid structure Biol Chem 2002 383 945 960 10.1515/BC.2002.101 12222684

[B37] McLeod SM Johnson RC Control of transcription by nucleoid proteins Curr Opin Microbiol 2001 4 152 159 10.1016/S1369-5274(00)00181-8 11282470

[B38] Bonnefoy E Rouviére-Yaniv J HU and IHF, two homologous histone-like proteins of *Escherichia coli*, form different protein-DNA complexes with short DNA fragments EMBO J 1991 10 687 696 2001682 10.1002/j.1460-2075.1991.tb07998.x PMC452703

[B39] Freundlich M Ramani N Mathew E Sirko A Tsui P The role of integration host factor in gene expression in *Escherichia coli* Mol Microbiol 1992 6 2557 2563 10.1111/j.1365-2958.1992.tb01432.x 1447969

[B40] Dillon SC Dorman CJ Bacterial nucleoid-associated proteins, nucleoid structure and gene expression Nat Rev Microbiol 2010 8 185 195 10.1038/nrmicro2261 20140026

[B41] Hales LM Gumport RI Gardner JF Examining the contribution of a dA+dT element to the conformation of *Escherichia coli *integration host factor-DNA complexes Nucleic Acids Res 1996 24 1780 1786 10.1093/nar/24.9.1780 8650000 PMC145845

[B42] Goosen N Van de putte P The regulation of transcription initiation by integration host factor Mol Microbiol 1995 16 1 7 10.1111/j.1365-2958.1995.tb02386.x 7651128

[B43] Dorman CJ H-NS: a universal regulator for a dynamic genome Nat Rev Microbiol 2004 2 391 400 10.1038/nrmicro883 15100692

[B44] Cotter PA Miller JF *In vivo *and *ex vivo *regulation of bacterial virulence gene expression Curr Opin Microbio 1998 1 17 26 10.1016/S1369-5274(98)80138-0 10066465

[B45] Friedberg D Umanski T Fang Y Rosenshine I Hierarchy in the expression of the locus of enterocyte effacement genes of enteropathogenic *Escherichia coli* Mol Microbiol 1999 34 941 952 10.1046/j.1365-2958.1999.01655.x 10594820

[B46] Dorman CJ Regulatory integration of horizontally-transferred genes in bacteria Front Biosci 2009 14 4103 4112 19273337 10.2741/3515

[B47] Lercher MJ Pál C Integration of horizontally transferred genes into regulatory interaction networks takes many million years Mol Biol Evol 2008 25 559 567 10.1093/molbev/msm283 18158322

[B48] Sambrook J Fritsch EF Maniatis T Molecular cloning: a laboratory manual 1989 2 Cold Spring Harbor. New York

[B49] Chen WP Kuo TT A simple and rapid method for the preparation of gram negative bacterial genomic DNA Nucleic Acids Res 1993 21 2260 10.1093/nar/21.9.2260 8502576 PMC309503

[B50] Rowley KB Clements DE Mnadel M Humphrey T Patil SS Multiple copies of a DNA sequence from *Pseudomonas syringae *pathovar *phaseolicola *abolish thermoregulation of phaseolotoxin production Mol Microbiol 1993 8 625 635 10.1111/j.1365-2958.1993.tb01606.x 8326870

[B51] Bradford MM A rapid and sensitive method for the quantitation of microgram quantities of protein utilizing the principle of protein-dye binding Anal Biochem 1976 72 248 254 10.1016/0003-2697(76)90527-3 942051

[B52] Demczuk S Harbers M Vennstrom B Identification and analysis of all components of a gel retardation assay by combination with immunoblotting Proc Natl Acad Sci USA 1993 90 2574 2578 10.1073/pnas.90.7.2574 8385336 PMC46137

[B53] Joardar V Lindeberg M Jackson RW Selengut J Dodson R Brinkac LM Daugherty SC DeBoy R Durkin AS Giglio MG Madupu R Nelson WC Rasovitz MJ Sullivan S Crabtree J Creasy T Davidsen T Haft DH Zafar N Zhou L Halpin R Holley T Khouri H Feldblyum T White O Fraser CM Chatterjee AK Cartinhour S Schneider DJ Mansfield J Collmer A Buell R Whole genome sequence analysis of *Pseudomonas syringae *pv phaseolicola 1448A reveals divergence among pathovars in genes involved in virulence and transposition J Bacteriol 2005 187 6488 6498 10.1128/JB.187.18.6488-6498.2005 16159782 PMC1236638

