# Supplementary material for: Integration Host Factor (IHF) binds to the promoter region of the phtD operon involved in phaseolotoxin synthesis in P. syringae pv. phaseolicola NPS3121
Source: BMC Microbiol. 2011 May 4;11:90. doi: 10.1186/1471-2180-11-90 (PMC3112066; doi:10.1186/1471-2180-11-90)
Supplement: Additional file 1 — In this Power Point file we show the results of gel shift assays with the protein extracts of P. syringae pv. phaseolicola NPS3121 grown at 28°C and 18°C, as well as the supershift assays using unrelated antibodies, including anti-His, anti-GST, and anti Rlk. Furthermore, we show the gel competition assays using the algD promoter as competitor. Detection of binding to PphtD in extracts of P. syringae pv. phaseolicola NPS3121. Gel shift assays was performed using a radiolabeled PphtD fragment (-111 to +188) and crude extracts of P. syringae pv. phaseolicola NPS3121 grown at 18°C and 28°C in M9 minimal medium. Probe concentration was 0.05 pmol and protein concentration of crude extracts in each reaction was as follows: lane 1, no protein; lanes 2 and 3, 30 g. DNA-protein complex is indicated by an arrow. Supershift assays using unrelated antibodies. The assays were carried out using unrelated antibodies, including anti-His, anti-GST (both commercially available), and anti-Rlk, which validated the specificity of the anti-DNABII antibody. Furthermore, we show control experiments in which the DNA probe was mixed with the DNA-BII antibody in the absence of protein extract. The retarded and super-retarded complexes are indicated by an arrow. Gel shift competition assays with the algD promoter. Panel A shows the competition assays using the algD promoter region (500 bp), which includes the IHF binding site reported by Wozniak [32] as competitor. Competitors were added in increasing concentrations: 50 ng (0.15 pmol), 60 ng (0.18 pmol), 100 ng (0.3 pmol), 150 ng (0.45 pmol), 200 ng (0.6 pmol), and 300 ng (0.9 pmol). Panel B depicts the competition assays with the algD promoter region (265 bp) that does not contain the IHF binding site. The competitor concentration used was: 50 ng (0.29 pmol), 60 ng (0.34 pmol), 100 ng (0.57 pmol), 150 ng (0.86 pmol), 200 ng (1.14 pmol), and 300 ng (1.72 pmol). [file 1471-2180-11-90-S1.PPT]

## Slide 1
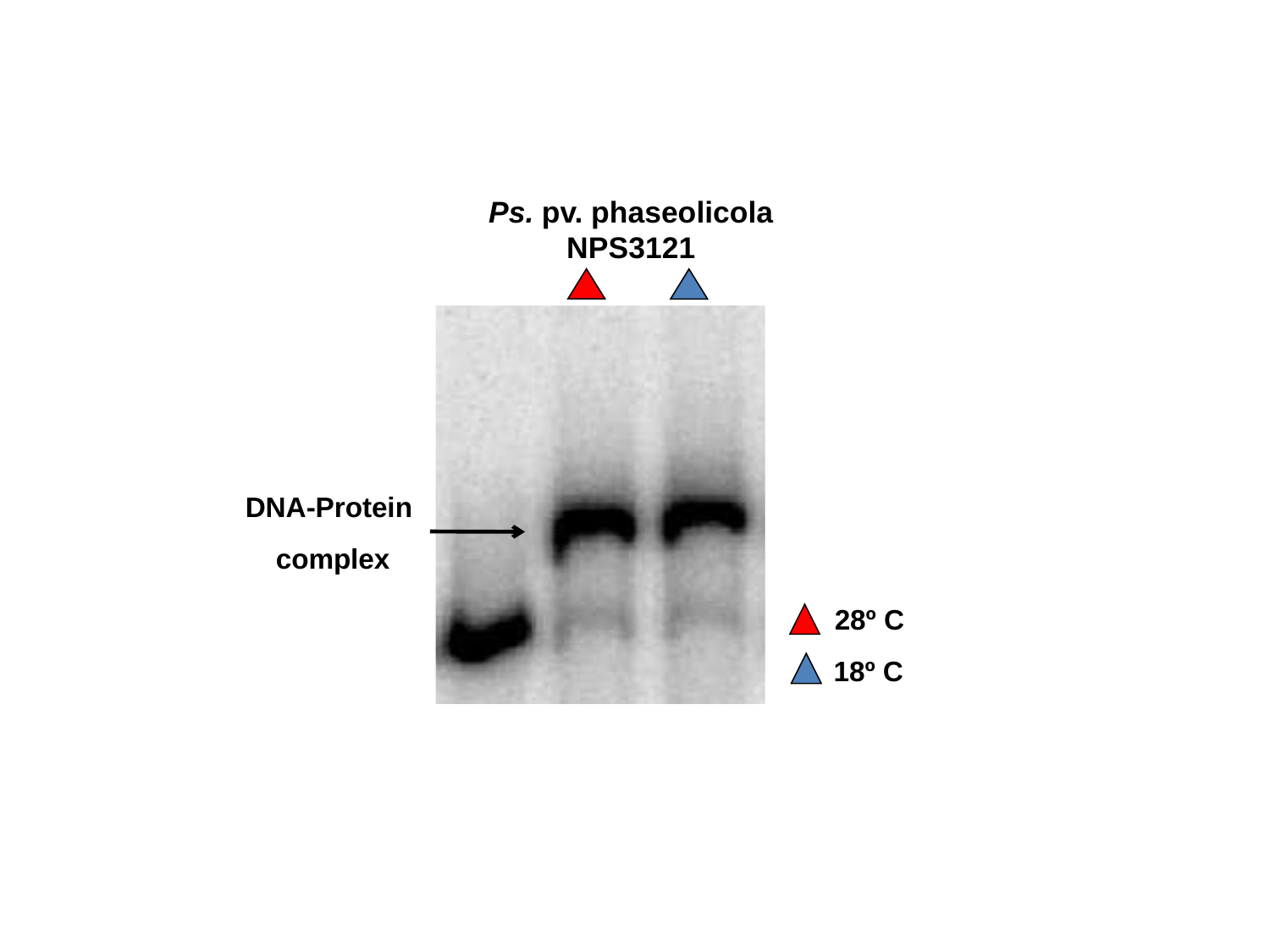

Ps. pv. phaseolicola NPS3121
DNA-Protein
complex
28º C
18º C

## Slide 2
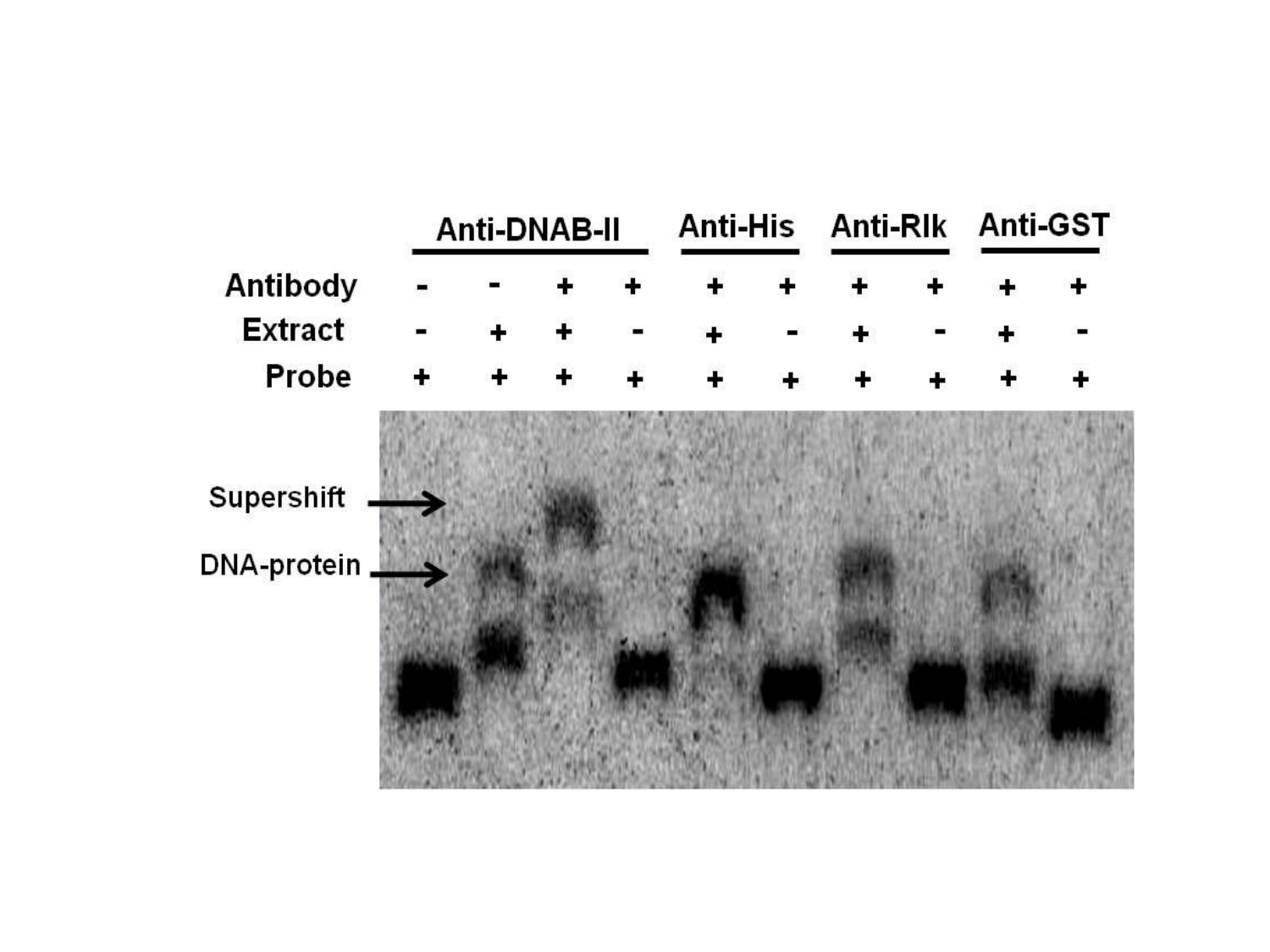

## Slide 3
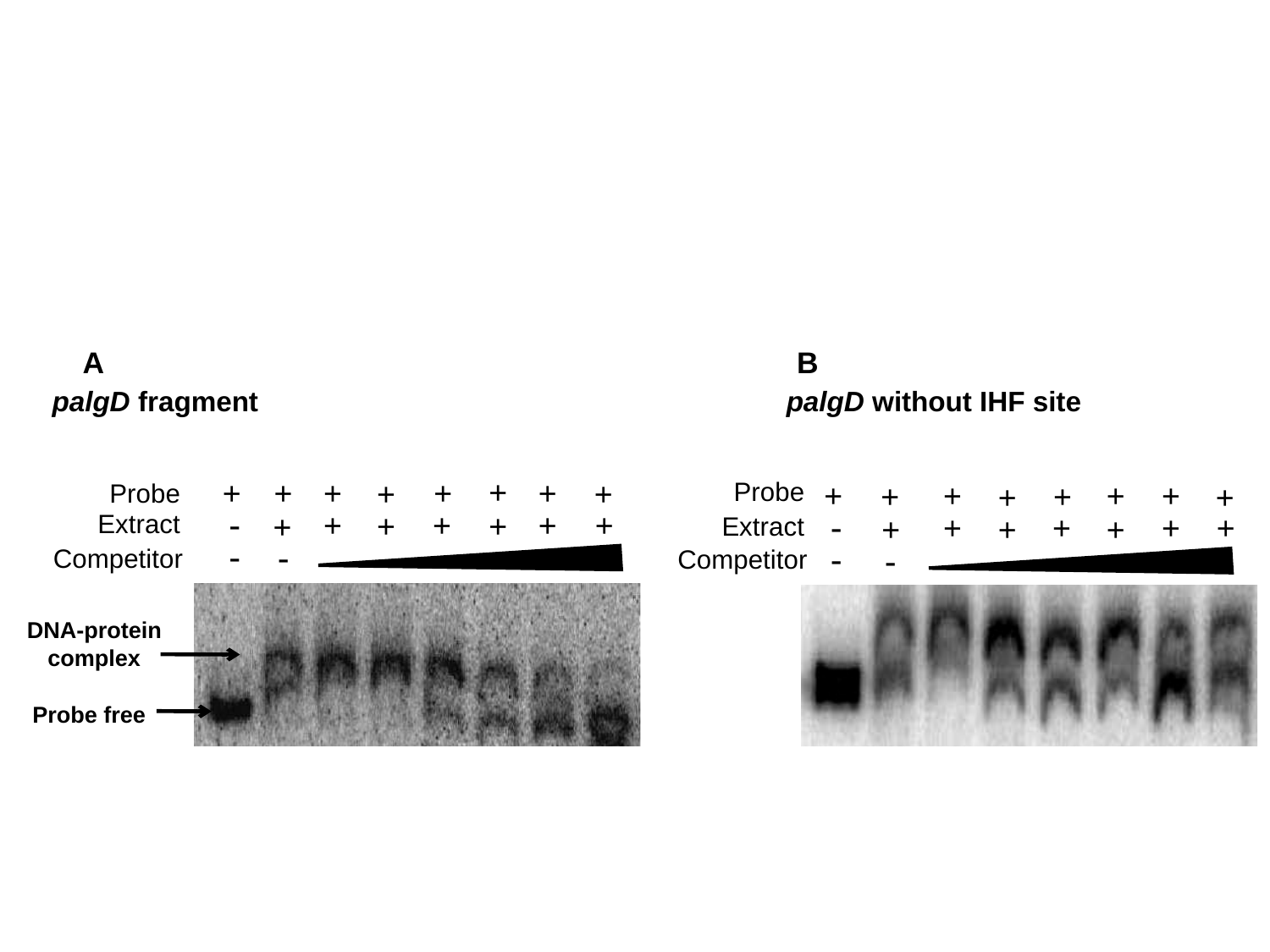

A
B
palgD fragment
palgD without IHF site
+
+
+
+
+
+
+
+
Probe
+
+
+
+
+
+
+
+
Probe
-
+
+
+
+
-
+
+
+
Extract
+
+
+
+
+
+
+
Extract
-
-
-
-
Competitor
Competitor
DNA-protein complex
Probe free
