# Supplementary material for: Integration Host Factor (IHF) binds to the promoter region of the phtD operon involved in phaseolotoxin synthesis in P. syringae pv. phaseolicola NPS3121
Source: BMC Microbiol. 2011 May 4;11:90. doi: 10.1186/1471-2180-11-90 (PMC3112066; doi:10.1186/1471-2180-11-90)
Supplement: Additional file 2 — This Word file contains tables listing the strains and plasmids used in this study, as well as the sequence of oligonucleotides and probes used in gel shift assays. [file 1471-2180-11-90-S2.DOC]

**Table 1.** Bacterial strains and plasmids used

| **Strain or plasmid** | **Relevant characteristics^a^** | **Reference(s) or source** |
| --- | --- | --- |
| Bacterial Strains |  |  |
|  |  |  |
| *Pseudomonas syringae* |  |  |
| pv. phaseolicola NPS3121 | Wild type, Toxigenic, Rf^r^ | [1] |
| pv. phaseolicola CLY233 | Non- toxigenic, does not contain *pht* region | [2] |
| pv. tomato DC3000 | Wild type, coronatine producing | [3] |
|  |  |  |
|  |  |  |
| *Escherichia coli* |  |  |
| TOP 10 | Commercial strain used for cloning | Invitrogen |
| JM103 |  | [4] |
| K12 substr. MG1655 | F- lambda- *ilvG*- *rfb*-50 *rph*-1 | [5] |
| K12 substr. MG1655 *ihf*A^-^ | *ihfA* gene deleted | [5] |
| K12 substr. MG1655 *ihf*B^-^ | *ihfB* gene deleted | [5] |
| K12 substr. MG1655 *hup*A^-^ | *hupA* gene deleted | [5] |
| K12 substr. MG1655 *hup*B^-^ | *hupB* gene deleted | [5] |
|  |  |  |
| Plasmids |  |  |
| pCR4Blunt-TOPO | Ap^r^ Km^r^, 3.95kb | Invitrogen |
| pUA66 | Low-copy number plasmid, with pSC101 origin, Km^r^, contains *gfpmut2* reporter gene; 4.26kb | [6] |
| pUC19 | Ap^r^, 2.69kb; *lac Z´* | Invitrogen |
| pP*ihfA* | pCR4-TOPO with alpha subunit gen of IHF of *P. syringae* pv. phaseolicola NPS3121; Km^r^ Ap^r^ | This study |
| pJLAG | pUA66; 416-bp fragment corresponding intergenic region *phtC-phtD*. Km^r^. | This study |

^a^ Ap^r^, Km^r^, and Rf^r^: resistance to ampicilin, kanamycin, and rifampin respectively.

**Table 2.** PCR primers designed and tested in this study

| **Primers** | **Sequence (5´-3´)**^a^ |
| --- | --- |
| L100074 | CCCTAGCTGGACGTGGAATC |
| L100075 | GCCCCGAGAAAGTCTTGTGC |
| L100137phtDr | CTGGATTGCAGCTGGCTTATCGAGCGG |
| L100150phtD-R | AACATCATGCAACAATTTATCTGAA |
| L100161phtD1Dr | GCGCCGAAAACTTCCAGCTGACATAAG |
| L100194phtD | GATAAATTGTTGCATGATGTTTGTT |
| L100195revphtD | CTTATCGAGCGGAGCCACTTAAACC |
| L100200 | TAGAGCTGTTTGATTTTATTTTTCAG |
| L100216palgDf | GTAGCACTTTCATATCCACCCCGCT |
| L100217palgDr | CTGACGCCAGCGTTACATGAACCTG |
| L100255phtDr | CCACTTAAACCTAACAGCAGAACAAAC |
| L100258ihfA | GCTTGAGGAGAGGTTAAACGCCACG |
| L100259ihfA*Bam*HI | GAAGTAGCGGATCCCGGGGATCGGCGGTAGCTC |
| L100269FphtD*Xho*I | AGTGCCCTCTCGAGTTGCCTGGTTAATAC |
| L100270phtD*Bam*HI | CACTGACGGGATCCAACTTCATTGAGGA |
| L100270palgDf | AAATGCCAACATCGTGACGCCAGAT |
| L100271palgDr | TTCGGAAAGCCTCCGGCTAGAGCCT |

^a^ Sites for restriction enzymes are underlined

**Table 3. Probes used in gel mobility shift assays**

| **Probe** | **Primers** | **Positions of forward and reverse primers^a^** | **Amplicon length (bp)** |
| --- | --- | --- | --- |
| A | L100074-L100075 | -111 to +188 | 300 |
| B | L100074-L100161 | -111 to +90 | 202 |
| C | L100074-L100137 | -111 to +22 | 133 |
| D | L100074-L100195 | -111 to +6 | 118 |
| E | L100194-L100195 | -52 to +6 | 59 |
| F | L100194-L100075 | -52 to +188 | 241 |
| G | L100074-L100150 | -111 to -31 | 80 |
| H | L100200-L100255 | -77 to -8 | 70 |
| I | L100074-L100255 | -111 to -8 | 104 |

^a^ Positions with respect to the transcription start site of the *phtD* operon

**Table 4.** Probe oligonucleotides with mutations in proposed IHF site

| **Primer** | **Sequence (5´-3´)**^a^ |
| --- | --- |
| L100271 | CCCTAGCTGGACGTGGAATCATTTTGATATCGCATAGAGCTGTTTGA**GGGGCGGGCCA**A**T**ATAAATTGTTGCATGATGTTTGTTCTGCTGTTAGGTTTAAGTGG |
| L100272 | CCACTTAAACCTAACAGCAGAACAAACATCATGCAACAATTTAT**A**T**TGGCCCGCCCC**TCAAACAGCTCTATGCGATATCAAAATGATTCCACGTCCAGCTAGGG |
| L100275 | CCCTAGCTGGACGTGGAATCATTTTGATATCGCATAGAGCTGTTTGA**GGGGCGGGCCA**A**T**AT**GGGCCTGGA**CATGATTTTTGTTCTGCTATTAGGTTTAAGTGG |
| L100276 | CCACTTAAACCTAATAGCAGAACAAAAATCATG**TCCAGGCCC**AT**A**T**TGGCCCGCCCC**TCAAACAGCTCTATGCGATATCAAAATGATTCCACGTCCAGCTAGGG |

^a^ The bold red letters corresponding to the mutated bases

**REFERENCES**

1. Peet RC, Lindgren PB, Willis DK, Panopoulos NJ: **Identification and cloning of genes involved in phaseolotoxin production by *Pseudomonas syringae* pv. “phaseolicola”.** *J Bacteriol* 1986, **166**:1096-1105.
2. Oguiza JA, Rico A, Rivas LA, Sutra L, Vivian A, Murillo J: ***Pseudomonas syringae* pv. phaseolicola can be separated into two genetic lineages distinguished by the possession of the phaseolotoxin biosynthetic cluster.** *Microbiology* 2004, **150**:473-482.
3. Buell CR, Joardar V, Lindeberg M, Selengut J, Paulsen IT, Gwinn ML, Dodson RJ, Deboy RT, Durkin AS, Kolonay JF, Madupu R, Daugherty S, Brinkac L, Beanan MJ, Haft DH, Nelson WC, Davidsen T, Zafar N, Zhou L, Liu J, Yuan Q, Khouri H, Fedorova N, Tran B, Russell D, Berry K, Utterback T, Van-Aken SE, Feldblyum TV, D'Ascenzo M, Deng WL, Ramos AR, Alfano JR, Cartinhour S, Chatterjee AK, Delaney TP, Lazarowitz SG, Martin GB, Schneider DJ, Tang X, Bender CL, White O, Fraser CM, Collmer A: **The complete genome sequence of the *Arabidopsis* and tomato pathogen *Pseudomonas syringae* pv. tomato DC3000.** *Proc Natl Acad Sci USA* 2003, **100**:10181-10186.
4. Yanish-Perron C, Vieira J, Messing J: **Improved M13 phage cloning vectors and host strains: nucleotide sequences of the M13mp18 and pUC19 vectors.** *Gene* 1985, **33**:103-19.
5. Baba T, Ara T, Hasegawa M, Takai Y, Okumura Y, Baba M, Datsenko KA, Tomita M, Wanner BL, Mori H: **Construction of *Escherichia coli* K-12 in frame, single-gene knockout mutants: the Keio collection.** *Mol Syst Biol*, 2:2006.0008.
6. Zaslaver A, Bren A, Ronen M, Itzkovitz S, Kikoin I, Shavit S, Liebermeister W, Surette MG, Alon U: **A comprehensive library of fluorescent transcriptional reporters for *Escherichia coli.*** *Nat Methods* 2006, **3**:623-628.
